# Study protocol: high-dose mizoribine with prednisolone therapy in short-term relapsing steroid-sensitive nephrotic syndrome to prevent frequent relapse (JSKDC05 trial)

**DOI:** 10.1186/s12882-018-1033-z

**Published:** 2018-09-10

**Authors:** Taketsugu Hama, Koichi Nakanishi, Kenji Ishikura, Shuichi Ito, Hidefumi Nakamura, Mayumi Sako, Mari Saito-Oba, Kandai Nozu, Yuko Shima, Kazumoto Iijima, Norishige Yoshikawa, for the Japanese Study Group of Kidney Disease in Children (JSKDC)

**Affiliations:** 1 Department of Pediatrics, Wakayama Medical University, Wakayama, Japan; 2 Department of Child Health and Welfare (Pediatrics), Graduate School of Medicine, University of the Ryukyus, 207 Uehara, Nishihara, Okinawa 903-0215 Japan; 3 Division of Nephrology and Rheumatology, National Center for Child Health and Development, Setagaya-ku, Tokyo Japan; 4 Department of Pediatrics, Yokohama City University, Yokohama, Kanagawa Japan; 5 Clinical Research Center, National Center for Child Health and Development, Setagaya-ku, Tokyo Japan; 6 Division of Clinical Trials, Department of Clinical Research Promotion, Clinical Research Center, National Center for Child Health and Development, Setagaya-ku, Tokyo Japan; 7 Department of Medical Statistics, Toho University School of Medicine, Ota-ku, Tokyo Japan; 8 Department of Pediatrics, Kobe University Graduate School of Medicine, Kobe, Hyogo Japan; 9 Clinical Research Center, Wakayama Medical University, Wakayama, Japan

**Keywords:** Mizoribine, Frequently-relapsing nephrotic syndrome, Steroid-sensitive nephrotic syndrome

## Abstract

**Background:**

Eighty percent of children with steroid-sensitive nephrotic syndrome (SSNS) relapse within 2 years and 40–50% patients show frequently-relapsing nephrotic syndrome (FRNS). Patients showing a relapse within 6 months after initial remission are at high risk of FRNS. Since frequent prednisolone treatment for FRNS induces severe prednisolone side effects, development of a treatment to prevent patients from shifting to FRNS is desirable. Mizoribine is an immunosuppressive drug with fewer side effects than prednisolone. Recent studies reported the efficacy of high-dose mizoribine in children with FRNS.

**Methods/design:**

We conduct a multicenter, open, randomized controlled trial to investigate the efficacy and safety of standard prednisolone plus high-dose mizoribine therapy in children with SSNS showing a relapse within 6 months after an initial remission. Patients are allocated to either standard prednisolone alone treatment group, or standard prednisolone plus high-dose mizoribine group. For the former group, mizoribine is administered at a dose of 10 mg/kg/day once daily and continued for 2 years. The primary endpoint is the duration to frequent relapse.

**Discussion:**

The results provide important data on use of high-dose mizoribine to prevent SSNS patients from shifting to FRNS. Since blood concentrations of mizoribine have not been investigated in detail until now, there is a possibility that mizoribine is underestimated in favor of other immunosuppressive drugs. In future, high-dose mizoribine therapy may lead to prevention of relapse in children at high risk of FRNS, and to decreased total dose of prednisolone.

**Trial registration:**

UMIN000005103, (Prospectively registered 1st March 2011).

## Background

Prednisolone (PSL) is the first-line drug for the treatment of idiopathic nephrotic syndrome (NS) in children. Ninety percent of patients with childhood NS have steroid-sensitive nephrotic syndrome (SSNS) and remit within 4 weeks after initiation of PSL therapy. After remission, patients require no drug treatment until relapse. Approximately 80% of patients with SSNS relapse within 2 years of PSL treatment as part of the International Study of Kidney Disease in Children (ISKDC) regimen. Around 40 to 50% of patients shift to either “frequently-relapsing NS (FRNS)” as repeating relapse in a relatively short period, or “steroid-dependent NS (SDNS)” as relapsing with decreasing or stopping PSL [1].

Frequent PSL treatment becomes problematic because of the side effects of steroids in FRNS patients. It is clear that patients who relapse immediately (within 6 months) after remission by PSL treatment for the first episode of NS tend to shift to FRNS [2]. It is therefore desirable to inhibit patients from relapsing soon after remission and to avoid the shift to FRNS.

Mizoribine is known to be safer than other immunosuppressive drugs due to its pharmacological characteristics [3, 4]. A placebo-controlled randomized trial in children with FRNS demonstrated that mizoribine has an inhibitory effect for relapsing only in patients 10 years old or younger [3]. Conventional use of mizoribine (3–4 mg/kg daily) leads to a peak blood level at around 1.0 μg/mL [5] although a peak blood level of 2.5–3.0 μg/mL is needed to sustain long-term efficacy for treatment of glomerular diseases [6, 7]. It is thought that children need to take double the dosage of mizoribine as adults because the volume of extracellular fluid of children is larger than in adults and interstitial drug absorption efficacy of children might be lower [8–11].

Recently, high-dose mizoribine administrations aimed to keep high serum mizoribine concentration have been considered for children with FRNS [12, 13]. Serum concentration after 2 h of mizoribine administration (C2) was about 3 μg/mL in patients who achieved decrease in the number of relapses. These patients took 10 mg/kg/day of mizoribine once a day, and there were no severe side effects.

Patients with NS under 10 years old and who relapse within 6 months after remission for the first episode of NS are considered to be high risk of FRSN [2]. We therefore focus on the possibility that adding high-dose mizoribine to the standard PSL treatment for SSNS patients who are likely to shift to FRNS could prevent them from shifting to FRNS. Here, we conduct a multicenter open randomized controlled trial to evaluate the efficacy and safety of PSL plus high-dose mizoribine treatment.

## Methods/design

### Aim

This trial aims to investigate whether standard PSL plus high-dose mizoribine combination therapy is superior to standard PSL alone in preventing a shift to FRNS for children with SSNS who immediately (within 6 months) relapse after remission with the ISKDC PSL treatment for the first episode of NS.

### Study design and patients

**Fig. 1:**
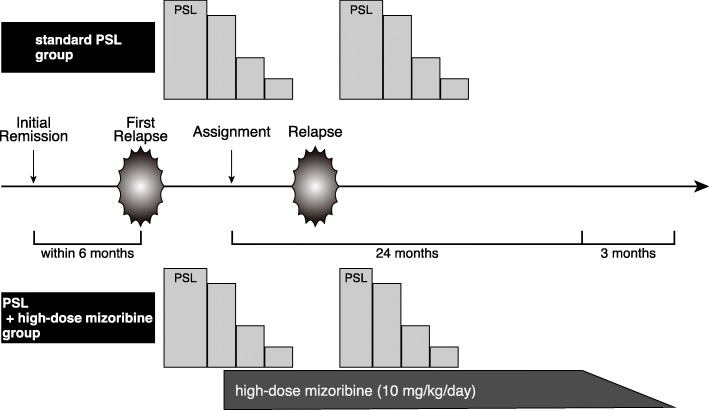
Treatment regimens

We conduct a multicenter, randomized, superiority, open-label trial and compare standard PSL plus high-dose mizoribine combination treatment with standard PSL alone treatment for children with SSNS who immediately (within 6 months) relapse after remission for the first episode of NS (Fig. 1). We diagnose NS and remission according to the ISKDC. Patients aged 2–10 years are eligible if they have remission within 3 weeks of PSL administration for the first relapse.

Inclusion and exclusion criteria are as follows:

- Inclusion criteria:
  1. Patients diagnosed with a first episode of NS who meet (a) urinary protein/creatinine ratio ≥ 1.8 and (b) ≤2.5 g/dL of serum albumin
  2. Patients treated by the ISKDC based regimen at the first episode and relapsed within 6 months after the first remission
  3. Age at the registration ≥2 and < 11 years
  4. Patients who remit within 3 weeks after PSL treatment for the first relapse
- Exclusion criteria:
  1. Patients diagnosed with nephritic NS
  2. Patients diagnosed with IgA vasculitis, systemic lupus nephritis or secondary NS
  3. Patients who have been diagnosed with steroid-resistant NS (SRNS)
  4. Patients who have been treated with any immunosuppressant for NS
  5. Patients with poorly controlled hypertension, impaired renal function, or severe liver dysfunction
  6. Patients who are participating in other studies at the time of registration.

### Randomization

Patients are randomly assigned to either the standard PSL plus high-dose mizoribine combination, or standard PSL alone group in a 1:1 ratio. We use a minimization method stratifying by age (2–5 years or 6–10 years), sex, duration from initial remission to relapse (< 90 days or ≥ 90 days) and institution as adjustment (stratification) factors. Patients, their guardians, treating physicians, and individuals assessing outcomes and analyzing data are not blinded to the patients’ treatment assignments. Apart from the trial statistician and the data-monitoring committee, all treating physicians and other investigators remain blinded to the trial results until follow-ups are completed.

### Procedures

For the standard PSL plus high-dose mizoribine group, mizoribine is administered at a dose of 10 mg/kg/day (maximum 300 mg/day) once daily after breakfast and continued for 2 years. Mizoribin is started within 7 days from the registration date. After 2-year administration or termination of this trial, dosage of mizoribine will be tapered every month and stopped within 3 months. In case of leukocytopenia or impaired renal function, mizoribine treatment will be tapered or stopped. PSL treatment for relapse during the observation period follows the ISKDC regimen.

The observation period is from the registration date to the completion or discontinuation date of this trial. Completion of study is defined as completing of 2-year administration. Patients who complete or discontinue this study will be followed-up.

### Outcomes

The primary endpoint is defined as the duration from registration to diagnosis of FRNS. Diagnosis of FRNS is based on relapse dates according to the ISKDC. In our study, FRNS is defined as two relapses within 6 months of initial remission, or four relapses within any 12-month period. This includes relapses during initial tapering treatment, but excludes relapses with spontaneous remissions. Relapse is defined as proteinuria 2+ or higher on dipstick analysis for 3 or more consecutive days or proteinuria 2+ or higher on dipstick analysis and serum albumin ≤2.5 g/dL. Immunosuppressant administration is prohibited in the protocol. If patients show SDNS or SRNS, they are treated as an event in the primary analysis. Data for patients who do not experience these events are considered censored at the last examination. Secondary endpoints are relapse-free period, the poportion of patients without relapses, the number of relapses per person/year, time to SDNS, time to SRNS, total prednisolone dose, and adverse events. Adverse events were recorded throughout the trial period and assessed using Common Terminology Criteria for Adverse Events v3.0. Serum mizoribine concentrations at trough and C3 are measured 1 month after administration of mizoribine to monitor safety and pharmacological effect.

### Statistical analyses

The primary analysis of this study is to examine the superiority of PSL plus high-dose mizoribine combination therapy compared to standard PSL alone therapy in the time to FRNS.

For the sample size determination, we based the hazard rate ratio (HR, the PSL plus high dose mizoribine treatment group to the PSL treatment group) on the two-year FRNS probability. We estimated 50% at two-year in the standard PSL treatment and postulated the HR of 0.50. The required sample size was calculated as 108 patients (54 per arm) under a two-sided alpha 5% and 80% power, if the patients accrued for 4 years and follow up for 2 years. Considering withdrawal of consent or loss, target sample number was determined as 120 cases (60 each). We used POWER procedure of SAS software (version 9.3) for this calculation.

The primary endpoint, time to FRNS will be compared between these two groups using log-rank test. Cox proportional hazard model is used to estimate the hazard ratio with a 95% confidence interval. Kaplan-Meier method is used to summarize time to FRNS. The statistical significance level is set to 5%.

Secondary endpoints of relapse-free period and duration shifting to SDNS or SRNS will be compared in a similar manner as primary endpoint. The proportion of patients without relapses will be compared by Fisher’s exact test. Number of relapse and total steroid dose will be analyzed by Wilcoxson test. Correlation between mizoribine dose and blood concentration of mizoribine will be evaluated by Spearman rank correlation coefficient analysis.

## Discussion

The purpose of this trial is to examine efficacy and safety of high-dose mizoribine in preventing patients with NS who show an early relapse within 6 months after initial remission from shifting to FRNS. One of most noteworthy adverse events in patients with SSNS is harmfulness of steroids, especially to those with FRNS/SDNS. It has been clarified that prolonged ISKDC steroid regimen for patients with SSNS did not shorten the relapse-free period [2, 14–16]. It is therefore reasonable to pursue new strategies to prevent patients with SSNS from shifting to FRNS. Although immunosuppressants are considered as second-step drugs next to steroids, use of them should be carefully decided due to severe side effects. Under such a condition, it is important to recognize predictive risk factors for FRNS. Previously, we clarified that a relapse within 6 months after initial treatment for NS is a predictive risk factor for FRNS [2]. Development of an optional drug regimen for patients who have such a risk factor is therefore desirable. From this perspective, we focused on mizoribine and conducted a randomized controlled trial to examine efficacy and safety of adding optional mizoribine to patients with NS.

Mizoribine has been developed in Japan. It is an imidazole-based nucleic acid-related substance derived from the culture fluid of *Eupenicillium brefeldianum.* Mizoribine is an antimetabolite to inhibit the purine synthesis pathway of nucleic acids, it shows an immunosuppressive effect of more selectively inhibiting lymphocyte proliferation [17, 18]. Unlike other immunosuppressive drugs, such as cyclosporine and cyclophosphamide, toxicity and myelosuppression of mizoribine seems to be milder. Mizoribine is also considered to have an effect to enhance glucocorticoid efficacy [19].

Historically, mizoribine has been used to suppress rejection reactions in kidney transplantation [20, 21], lupus nephritis [22, 23] and rheumatoid arthritis. Mizoribine has also been reported to be effective as a treatment for IgA nephropathy [24, 25]. It is approved as a treatment of NS in Japan [3, 26]. By adding mizoribine, the total dose of steroids can reportedly be reduced in treatment for NS. [27]. Furthermore, a double-blind, placebo-controlled, multicenter trial has shown that mizoribine significantly decreased relapse rate and prolonged remission period in children with FRNS in the subgroup consisting of patients 10 years old or younger [3]. Although efficacy and safety of mizoribine are well recognized in medical practice, blood concentration of mizoribine has not always been sufficient to reach efficacy. There is therefore a possibility that mizoribine is underestimated compared to other immunosuppressive drugs. Conventional use of mizoribine (3–4 mg/kg daily, maximum 150 mg/day) leads to a peak blood level at around 1.0 μg/mL [5], although a peak blood level of 2.5–3.0 μg/mL is biologically required to sustain long-term efficacy for the treatment of glomerular diseases [6, 7]. Consequently, high-dose mizoribine administrations aiming to retain high serum mizoribine concentration have come to be considered for children with FRNS [12, 13]. Serum C2 concentrations are known to be around 3 μg/mL in patients who achieved decrease in the number of relapses by taking 10 mg/kg/day of mizoribine once a day without severe side effects. Therefore, in the current protocol, we adopted dosage of 10 mg/kg/day of mizoribine once a day (maximum 300 mg/day).

If we can prevent at-risk patients from shifting to FRNS by adding high-dose mizoribine effectively and safely, we will reduce the total dose of steroids for patients, resulting in less side effects. Moreover, we will avoid use of other immunosuppressants, such as cyclosporine and cyclophosphamide, which are proven to be more effective but have more serious side effects than mizoribine. Patients with mizoribine treatment do not require frequent blood tests or renal biopsy, which is seen as a great benefit. As a result, it is expected to reduce medical expenses despite cost of mizoribine.

## Summary

The present study aims to examine efficacy and safety of standard PSL plus high-dose mizoribine therapy used to prevent at-risk children with SSNS from shifting to FRNS. An early relapse within 6 months after initial remission in patients with SSNS is a definitive risk for the shift to FRNS. High-dose mizoribine administration promises biological and clinical bases for efficacy. These conditions give the protocol strong rationales. The results of this trial may be of great benefit to children with SSNS at risk of FRNS and to their families.

## Abbreviations

FRNS: frequently relapsing nephrotic syndrome
ISKDC: International Study of Kidney Disease in Children
NS: nephrotic syndrome
PSL: prednisolone
SSNS: steroid sensitive nephrotic syndrome
